# Editorial: Single Plant Cell Metabolomics

**DOI:** 10.3389/fpls.2020.00161

**Published:** 2020-03-02

**Authors:** Aleš Svatoš, Young Jin Lee, Zhibo Yang

**Affiliations:** ^1^ Research Group Mass Spectrometry/Proteomics, Max Planck Institute for Chemical Ecology Jena, Jena, Germany; ^2^ Department of Chemistry, Iowa State University, Ames, IA, United States; ^3^ Department of Chemistry & Biochemistry, University of Oklahoma, Norman, OK, United States

**Keywords:** plant, single cell, metabolism, *in situ*, mass spectrometry

This Research Topic portrays some current developments in plant single cell metabolic profiling/imaging and offers current technological solutions for a better understanding of plant biology/biochemistry at the single cell level. This Research Topic has progressed from being an emerging technology to one of the more innovative tools for probing metabolism in plants. From previous studies in single cell transcriptomics, it was clear that each cell type may show unique transcriptomic profiles that will ultimately result in differences in cell-specific metabolic signatures. Such metabolic signatures can be monitored at the single cell level and correlated with transcriptomics data for each single cell type. Metabolomics profiles might be altered upon stress (e.g., *via* an environmental change and mechanical damage), and an understanding of such metabolic changes can potentially, for example, lead to discovery of cures for plant diseases.

There are seven research articles in this Research Topic focusing on several aspects of single cell metabolic profiling and mass spectrometric imaging (MSI) of plant cells. These articles cover methods development, comparative study of different MSI technologies, and successful application of MSI to address cell specific metabolic heterogeneity. Studies herein were with either individual algal cells or whole plant tissues. The sample type analyzed mostly dictated choice of method. If individual cells were available, we can directly extract the metabolites using a special mechanical probe (Sun et al.) or a focused UV-laser beam (Baumeister et al.). Both approaches detected a similar set of metabolites that encompassed primary and secondary metabolites including diverse lipid classes. Of these two approaches, the laser-based method had higher throughput, as scanning with a laser is much faster than probing cells mechanically. However, sampling the exact cell of interest is not trivial when working with whole tissue. The Kertesz laboratory has suggested use of an automatic optics-guided laser capture microdissection (LCM) of individual cells with subsequent sample liquid vortex extraction followed by metabolite sampling and analysis using the mass spectrometer (Cahill and Kertesz). While this system is capable of handling several hundred cells from *Allium cepa* or cultured cells automatically, it works only on a monolayer of cells or a tissue cross-section, whereas multilayer and multicellular samples cannot be analyzed.

To examine the metabolic makeup of individual cells, the analysis should be done in a reasonable timeframe and under an undisturbed condition; it should be as close to the *in vivo* condition as possible and not postmortem. Matrix-assisted laser desorption ionization (MALDI) in atmospheric pressure (AP) condition has advantages in this regard compared to vacuum MALDI with much less fragmentation and the analysis of volatile compounds. However, as Li and colleagues show (Keller et al.) working under ambient conditions may not be without drawbacks. The authors tested two MALDI-based sources, a vacuum MALDI system fitted on an Orbitrap XL and an APMALDI source on Q-Exactive HF. About twice as many mass spectrometric features were observed in the vacuum system. While AP-MALDI may suffer from ion losses during ion transmission through its AP interface, it is not sufficient to claim the superiority of vacuum MALDI in general with this study alone. The authors proposed instrumental differences as a potential explanation such as in-source fragmentation, type of laser used, and ion transfer efficiencies.

The sensitivity of measurement can be augmented by chemical derivatization (Dueñas et al.) to enhance metabolite coverage. Applying Girard’s reagent for carbonyl groups, coniferyl aldehyde for amino groups, and 2-picoylamine for acid derivatizations directly on plant cross-sections increases the number of metabolomics features to ca 600 additional (new) ones as compared to a nonderivatized sample. In future, specific chemotypes present in minute amounts can be highlighted by reaction with a compound-class-specific reagent.

For all analytical methods, sample preparation is of principal importance. Minimal sample handling and reproducible metabolite extraction is a basic prerequisite for metabolomics studies. Two of the articles illustrate the potential solution of sample preparation by combining cell probing with secondary ionization by an electrospray (ESI) plume: electrospray laser desorption ionization (ELDI) and laser ablation electrospray ionization (LAESI). Both UV- (ELDI) or IR laser (LAESI) can be used to selectively desorb/ionize compounds of interest; see (McVey et al.) and (Stopka et al.), respectively. LAESI uses an IR laser tuned to the strongest absorbing region of water (2940 nm) and the overheated cellular water bursts open the plant cells. ELDI mostly desorbs UV-absorbing metabolites, and the uneven illumination of *S. scutellarioides* leaf halves is manifested by different profiles of phenolic compounds. LAESI seems to be more generally applicable and a very rich mixture of both primary and secondary metabolites could be visualized. In general, use of UV in LDI or MALDI provides higher spatial resolution than use of IR. For example, 10 μm spatial resolution was demonstrated here for both vacuum (Dueñas et al.) and AP (Baumeister et al.). Use of IR in LAESI typically has a limited spatial resolution of ~200 μm; however, Stopka et al. adopted fiber- based LAESI (f-LAESI) to achieve 15 μm spatial resolution and access metabolic profiles of specific cell types by combining this with fluorescence and bright-field microscopy. In this way, subpopulations of epidermal cells and excretory idioblasts of *Egeria densa* leaf blade were studied. In the current report of UV-LDI combined with ESI post-ionization, ELDI (McVey et al.), the spatial resolution was limited to 125 μm, which is not quite at the single cell level and further improvement will be necessary.

Overall, additional new innovations are needed to fully support single cell biology/biochemical analyses. No absolute quantification of metabolites is currently available and the identification of metabolites by tandem mass spectrometry or other methods (Wolfender et al., 2019) must be further developed. In spite of such efforts, characterization of full chemical structures (e.g., structural isomers and stereoisomers) remains a formidable challenge, especially in single cell analysis, and should be taken into account in provisional annotation of the metabolites and the accompanying biological interpretations.

## Author Contribution

AS wrote the text, YJL and ZY edited the text, all authors approve the submission.

## Conflict of Interest

The authors declare that the research was conducted in the absence of any commercial or financial relationships that could be construed as a potential conflict of interest.
